# Supplementary material for: Polyketide synthase gene clusters in the ericoid mycorrhizal fungus Oidiodendron maius: genome-wide prediction, expression profiling, and functional characterization of knockout mutants
Source: Front Plant Sci. 2026 Sep 16;17:1924430. doi: 10.3389/fpls.2026.1924430 (PMC13623903; doi:10.3389/fpls.2026.1924430)
Supplement: Supplementary file 1 [file SupplementaryFile1.zip › Supplementary Materials/Supplementary Folder 1.zip!/Oidma1_AssemblyScaffolds_Repeatmasked/knownclusterblast/cluster35/jgi.p_Oidma1_32791_mibig_hits.html]

| MIBiG Protein | Description | MIBiG Cluster | MiBiG Product | % ID | % Coverage | BLAST Score | E-value |
| --- | --- | --- | --- | --- | --- | --- | --- |
| CBF70385.1 | metallo-beta-lactamase\_domain\_protein\_(AFU\_orthologue;\_AFUA\_7G00120) | BGC0000684 | Asperthecin\_biosynthetic\_gene\_cluster | 69 | 98.4126984127 | 458 | 4.7e-129 |
| AEN83888.1 | AdaB | BGC0000156 | TAN-1612\_biosynthetic\_gene\_cluster | 68 | 97.4603174603 | 446 | 3.2e-125 |
| EGD99351.1 | metallo-beta-lactamase | BGC0001144 | Neosartoricin\_biosynthetic\_gene\_cluster | 60 | 100.317460317 | 420 | 2.4e-117 |
| ADI24932.1 | VrtG | BGC0000168 | Viridicatumtoxin\_biosynthetic\_gene\_cluster | 64 | 97.1428571429 | 413 | 2.3e-115 |
| EAL89338.1 | metallo-beta-lactamase\_domain\_protein,\_putative | BGC0001403 | Trypacidin\_biosynthetic\_gene\_cluster | 48 | 100.0 | 312 | 7.1e-85 |
| ADM34149.1 | metallo-beta-lactamase\_domain\_protein | BGC0001084 | Notoamide\_/\_stephacidin\_biosynthetic\_gene\_cluster | 49 | 94.9206349206 | 304 | 1.5e-82 |
| AGO59041.1 | PtaB | BGC0000121 | Pestheic\_acid\_biosynthetic\_gene\_cluster | 49 | 93.6507936508 | 302 | 5.7e-82 |
| CBF90099.1 | metallo-beta-lactamase\_domain\_protein,\_putative\_(AFU\_orthologue;\_AFUA\_4G14570) | BGC0001271 | Emericellin\_biosynthetic\_gene\_cluster | 52 | 93.9682539683 | 300 | 2.2e-81 |
| EAL84396.1 | metallo-beta-lactamase\_domain\_protein | BGC0001118 | Endocrocin\_biosynthetic\_gene\_cluster | 46 | 94.9206349206 | 291 | 1e-78 |
| CBF79145.1 | conserved\_hypothetical\_protein | BGC0000013 | Alternariol\_biosynthetic\_gene\_cluster | 55 | 73.3333333333 | 281 | 1e-75 |
